# LacdiNAc synthase B4GALNT3 has a unique PA14 domain and suppresses *N*-glycan capping

**DOI:** 10.1016/j.jbc.2024.107450

**Published:** 2024-06-04

**Authors:** Yuko Tokoro, Masamichi Nagae, Miyako Nakano, Anne Harduin-Lepers, Yasuhiko Kizuka

**Affiliations:** 1 Institute for Glyco-core Research (iGCORE), Gifu University, Gifu, Japan; 2 Department of Molecular Immunology, Research Institute for Microbial Diseases, Osaka University, Suita, Japan; 3 Laboratory of Molecular Immunology, Immunology Frontier Research Center (IFReC), Osaka University, Suita, Japan; 4 Graduate School of Integrated Sciences for Life, Hiroshima University, Higashihiroshima, Japan; 5 University of Lille, CNRS, UMR 8576 -UGSF- Unité de Glycobiologie Structurale et Fonctionnelle, Lille, France

**Keywords:** B4GALNT3, glycobiology, glycoprotein biosynthesis, glycosylation, glycosyltransferase, LacdiNAc, *N*-linked glycosylation, PA14

## Abstract

Structural variation of *N*-glycans is essential for the regulation of glycoprotein functions. GalNAcβ1–4GlcNAc (LacdiNAc or LDN), a unique subterminal glycan structure synthesized by B4GALNT3 or B4GALNT4, is involved in the clearance of *N*-glycoproteins from the blood and maintenance of cell stemness. Such regulation of glycoprotein functions by LDN is largely different from that by the dominant subterminal structure, *N*-acetyllactosamine (Galβ1-4GlcNAc, LacNAc). However, the mechanisms by which B4GALNT activity is regulated and how LDN plays different roles from LacNAc remain unclear. Here, we found that B4GALNT3 and four have unique domain organization containing a noncatalytic PA14 domain, which is a putative glycan-binding module. A mutant lacking this domain dramatically decreases the activity toward various substrates, such as *N*-glycan, *O*-GalNAc glycan, and glycoproteins, indicating that this domain is essential for enzyme activity and forms part of the catalytic region. In addition, to clarify the mechanism underlying the functional differences between LDN and LacNAc, we examined the effects of LDN on the maturation of *N*-glycans, focusing on the related glycosyltransferases upstream and downstream of B4GALNT. We revealed that, unlike LacNAc synthesis, prior formation of bisecting GlcNAc in *N*-glycan almost completely inhibits LDN synthesis by B4GALNT3. Moreover, the presence of LDN negatively impacted the actions of many glycosyltransferases for terminal modifications, including sialylation, fucosylation, and human natural killer-1 synthesis. These findings demonstrate that LDN has significant impacts on *N*-glycan maturation in a completely different way from LacNAc, which could contribute to obtaining a comprehensive overview of the system regulating complex *N*-glycan biosynthesis.

Protein glycosylation is one of the most frequent post-translational modifications and plays essential roles in a huge variety of physiological processes, including development, immunity, fertilization, and learning (1, 2). In addition, dysregulation of glycosylation can cause disease development or aggravation. Glycans have enormous structural diversity, and even a slight alteration in glycan structure can significantly impact the pathology of various diseases, such as cancer, Alzheimer’s disease, diabetes, and muscular dystrophy (3, 4, 5), demonstrating the importance of maintaining the appropriate glycan profiles on proteins or in cells. Glycan profiles of certain proteins or cells are shaped by the sequential and competitive actions of glycosyltransferases in the endoplasmic reticulum and the Golgi apparatus (6). Almost all the enzymes responsible for glycan biosynthesis in humans have now been identified, and the active and silent glycan biosynthetic pathways in a certain cell can be roughly predicted based on RNA-Seq data (7). To more precisely predict and fine-tune the glycan structures in cells, an essential next step is to dissect how the activity of the glycosyltransferase for each step is regulated in cells.

GalNAcβ1–4GlcNAc (LacdiNAc or LDN) is a disaccharide structure found in *N*- and *O*-GalNAc glycans, and it is present at the subterminal or terminal position in glycans (8). The biosynthesis of LDN is catalyzed by either one of two isozymes, B4GALNT3 and B4GALNT4 (Fig. 1*A*) (9, 10). In many cases, *N*-acetyllactosamine (Galβ1-4GlcNAc, LacNAc) rather than LDN is present at the *N*-glycan subterminus, which is biosynthesized by B4GALTs and often further modified with terminal residues, such as sialic acid (Sia), fucose (Fuc), and glucuronic acid (GlcA) (11, 12). LDN and its sulfated form were originally discovered in *N*-glycans of a pituitary hormone (13), and later studies revealed that LDN is also expressed in several other glycoproteins, such as leukemia inhibitory factor receptor (14), integrin-β1 (15), and sclerostin (16), suggesting that LDN is selectively attached to limited proteins. Although recognition of the specific peptide motifs by B4GALNT3 and 4 was proposed (17, 18, 19), the detailed mechanism by which B4GALNT3 and 4 can act on limited glycoproteins in cells remains to be fully elucidated.

**Figure 1:**
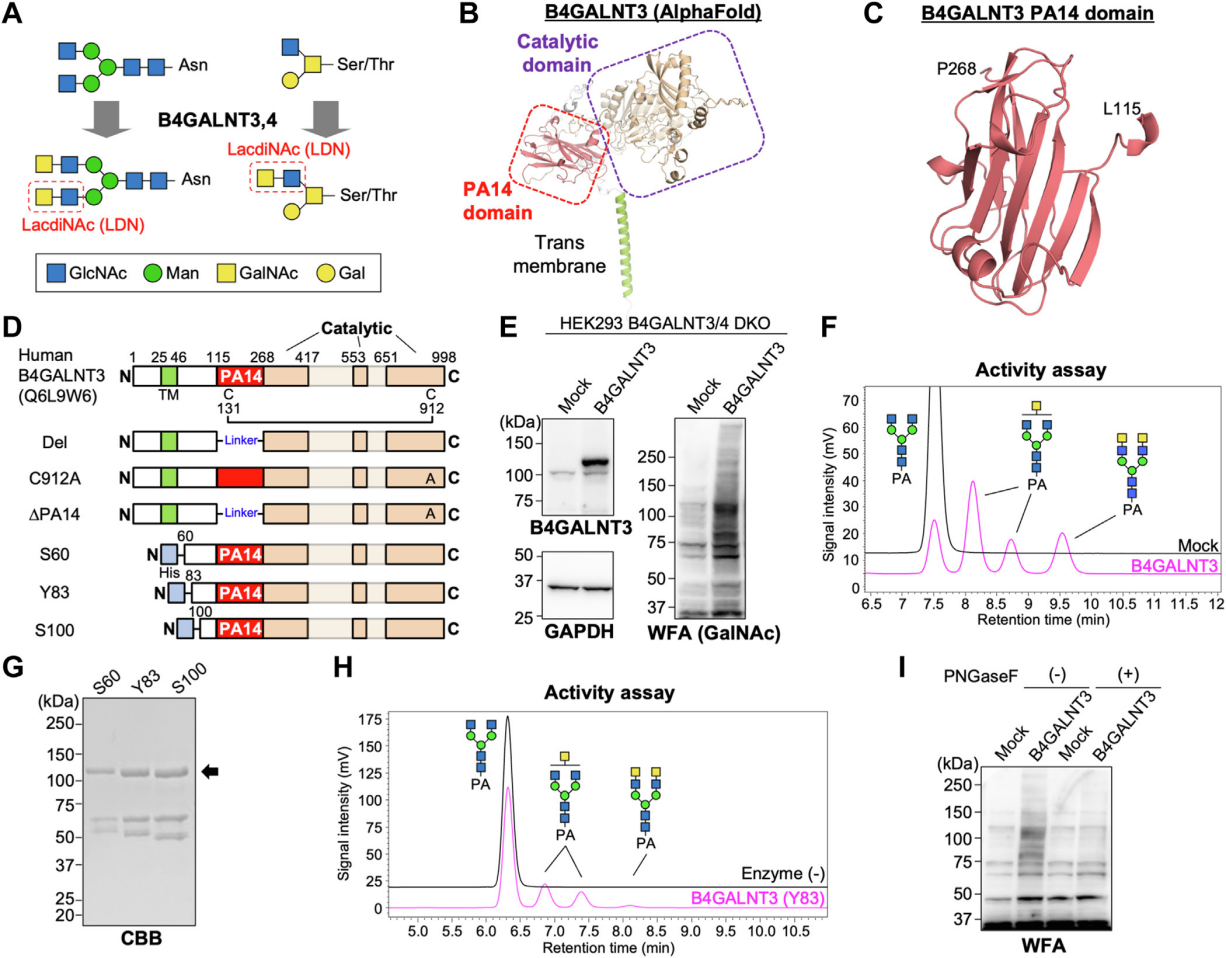
**Activity of LDN synthase, B4GALNT3, in cells and *in vitro* enzyme assay.***A*, schematic drawing of the actions of B4GALNT3 and 4. *B*, overall structure of human B4GALNT3 predicted by AlphaFold2. *C*, predicted structure of the PA14 domain of human B4GALNT3. *D*, domain architecture of membrane-spanning and soluble B4GALNT3 constructs. In Del and ΔPA14, the PA14 domain was replaced with GGGSGS linker. *E*, HEK293 cells deficient in B4GALNT3 and 4 (DKO) were transfected with the expression plasmid for B4GALNT3 or an empty vector (Mock). The cell lysates were blotted with anti-B4GALNT3, anti-GAPDH, and WFA. *F*, lysates of HEK293 DKO cells expressing B4GALNT3 were incubated with a PA-labeled acceptor glycan (GnGnbi-PA), and the reaction mixture was analyzed by reverse-phase HPLC. *G*, His-tagged truncated soluble forms of B4GALNT3 (S60, Y83, and S100) were expressed in HEK293T cells, purified from the medium through a Ni^2+^ column, and subjected to SDS-PAGE and CBB staining. *H*, purified B4GALNT3 Y83 was incubated with GnGnbi-PA and analyzed by reverse-phase HPLC. *I*, HEK293 DKO cells were transfected with the plasmid for B4GALNT3, and the lysates were incubated with PNGaseF and analyzed by blotting with WFA lectin. CBB, Coomassie brilliant blue; DKO, double KO; HEK293, human embryonic kidney 293 cell line; LDN, LacdiNAc; WFA, *Wisteria floribunda* agglutinin.

Regarding the biological functions of LDN, both physiological and pathological roles have been reported. Several circulating hormones were found to be modified with LDN, and their clearance from blood was shown to be accelerated by LDN modification (16, 20), in a manner mediated by asialoglycoprotein receptor and/or mannose receptor. Consequently, *B4galnt3*-KO mice exhibited an increased level of the bone morphogenic protein antagonist sclerostin in blood, resulting in a phenotype of accelerated bone loss (16). In addition, in humans, *B4GALNT3* variants are associated with lowered bone mineral density and higher risk of fractures (16). These reports suggest that LDN controls the levels of circulating hormones and bone mass. LDN has also been shown to be involved in the maintenance of cell stemness. For example, the knockdown of *B4GALNT3* in embryonic stem cells led to reduced self-renewal and proliferation because of dysfunction of the leukemia inhibitory factor receptor (14). Furthermore, silencing *B4GALNT3* also reduced cell stemness in colon cancer (21). Collectively, these findings underscore the biological significance of LDN, highlighting the importance of elucidating the molecular mechanism by which LDN exerts its functions and how LDN expression is regulated.

To understand the mechanisms regulating LDN expression, it is crucial to examine the mechanisms regulating its biosynthetic enzymes B4GALNT3 and 4. In general, various factors affect the activity of glycosyltransferases in cells, including their localization, shedding, and complex formation with other proteins (22, 23, 24). Substrate recognition by a noncatalytic domain was also recently revealed to be essential for the activity of certain glycosyltransferases. For instance, we recently found that *N*-glycan branching enzyme GnT-V (MGAT5) requires its N-domain for the recognition of glycoprotein substrates (25) and that other branching isozymes GnT-IVa and b (MGAT4A and MGAT4B) have a C-terminal lectin domain, which determines selectivity for substrate proteins (26). Furthermore, GALNTs, the initiation enzymes for *O*-GalNAc glycans, are also known to have a lectin domain for substrate recognition (27, 28, 29). The luminal stem domain of POMGNT1, the GlcNAc transferase for *O*-mannose (Man) glycans, has lectin activity and is suggested to recognize the substrate glycans or recruit an interaction partner to the specific *O*-Man glycans (30). In addition, fucosyltransferase FUT8 has an SH3 domain at the C terminus, which is required for acceptor glycan recognition (31, 32). B4GALNT3 and 4, like many other Golgi-resident glycosyltransferases, are type II membrane proteins comprising a short N-terminal cytosolic tail, a transmembrane region, and a large C-terminal luminal region containing a catalytic domain. By screening AlphaFold structures of all known human glycosyltransferases, we found that B4GALNTs have a unique noncatalytic domain directly connected to the catalytic domain (Fig. 1*B*). This β-sheet domain is designated as the PA14 domain, which is found in nonmammalian glycosidases and shown to be a carbohydrate-binding module (33, 34, 35). Although the role of the PA14 domain in the functions of the B4GALNTs is unknown, it is possibly involved in regulating the activity or substrate specificity of B4GALNTs.

As another probable function of LDN, we hypothesized that it affects global *N*-glycan profiles by suppressing or enhancing the actions of other related glycosyltransferases, particularly the enzymes acting on the *N*-glycan terminus. A recent study reported that simultaneous KO of B4GALNT3 and 4 in human embryonic kidney 293F (HEK293F) cells resulted in the loss of LDN as well as increased LacNAc and sialylation in *N*-glycans (36). Consistent with this, previous *in vitro* enzyme assays using disaccharides and trisaccharides showed that the major sialyltransferases for *N*-glycan, namely, ST6GAL1 and ST3GAL4, exhibited lower activity toward LDN than to LacNAc (37, 38, 39, 40). These findings imply that the replacement of common LacNAc with uncommon LDN significantly impacts the later steps in *N*-glycan biosynthesis, thereby regulating the glycoprotein functions. However, the relationships of LDN to other glycan epitopes in *N*-glycans and their enzymatic basis are largely unclear.

In this study, we aim to clarify how the unique PA14 domain of B4GALNT3 contributes to the enzymatic activity and how LDN biosynthesis affects later steps of *N*-glycan maturation. To this end, we generated a mutant lacking the PA14 domain and analyzed its activity. Furthermore, we examined the interplay between the biosynthesis of LDN and that of bisecting GlcNAc in *N*-glycan as well as the effects of the presence of LDN on the terminal modifications with Sia, Fuc, and human natural killer-1 (HNK-1). Our findings deepen our understanding of how complex *N*-glycan structures are biosynthesized in cells.

## Results

### B4GALNT3 has a unique PA14 domain

Structural prediction by AlphaFold2 showed that the human B4GALNTs has a PA14 domain in the luminal region (Fig. 1, *B* and *C*). The PA14 domain is found in many carbohydrate-related enzymes (34) and was demonstrated to recognize glycans in yeast adhesin (35) and possibly be involved in determining the substrate glycan specificity in a yeast glycosidase (33). On the basis of these findings, we hypothesized that the PA14 domain in B4GALNT3 plays an important role in its activity or substrate specificity. To investigate the roles of the PA14 domain, we constructed a plasmid for expressing a mutant B4GALNT3 lacking the whole of the PA14 domain (ΔPA14). To avoid undesired disulfide bonding when deleting PA14 domain, the cysteine in the C-terminal region (C912) of the mutant was also replaced with alanine in ΔPA14 mutant (Fig. 1*D*).

To examine the activity of WT and mutant B4GALNT3, we first established a system for detecting the intracellular and *in vitro* activity of B4GALNT3. Using HEK293 B4GALNT3 and B4GALNT4 double-KO (DKO) cells, we found that the overexpression of B4GALNT3 resulted in a drastic increase in the reactivity of GalNAc-recognizing lectin *Wisteria floribunda* agglutinin (WFA) (Fig. 1*E*) (41), indicating that the biosynthetic activity of B4GALNT3 in cells can be assessed using WFA. In addition, incubation of an acceptor substrate, fluorescence (2-aminopyridine, PA)-labeled GlcNAc-terminated biantennary *N*-glycan (GnGnbi-PA), with the cell lysates expressing B4GALNT3 as an enzyme source and UDP-GalNAc as a sugar donor generated reaction products that can be separated from the substrate by reverse-phase HPLC (Fig. 1*F*). The same reaction products were also observed when purified recombinant soluble B4GALNT3 was used as an enzyme (Fig. 1, *G* and *H*). We tested three soluble forms of B4GALNT3 (S60, Y83, and S100) (Fig. 1, *D* and *G*), and Y83 showed the highest activity. These findings indicated that this assay system can detect the *in vitro* enzyme activity of B4GALNT3. Furthermore, increased WFA reactivity by B4GALNT3 overexpression disappeared upon treating cell lysates with the *N*-glycan-removing enzyme PNGaseF in HEK293 DKO cells (Fig. 1*I*) and also in COS7 cells (Fig. S1*A*). These findings indicate that expressed B4GALNT3 is active toward *N*-glycans, and such activity in cells and *in vitro* reactions can be detected using our systems.

### PA14 domain is required for enzymatic activity of B4GALNT3 toward N-glycan

**Figure 2:**
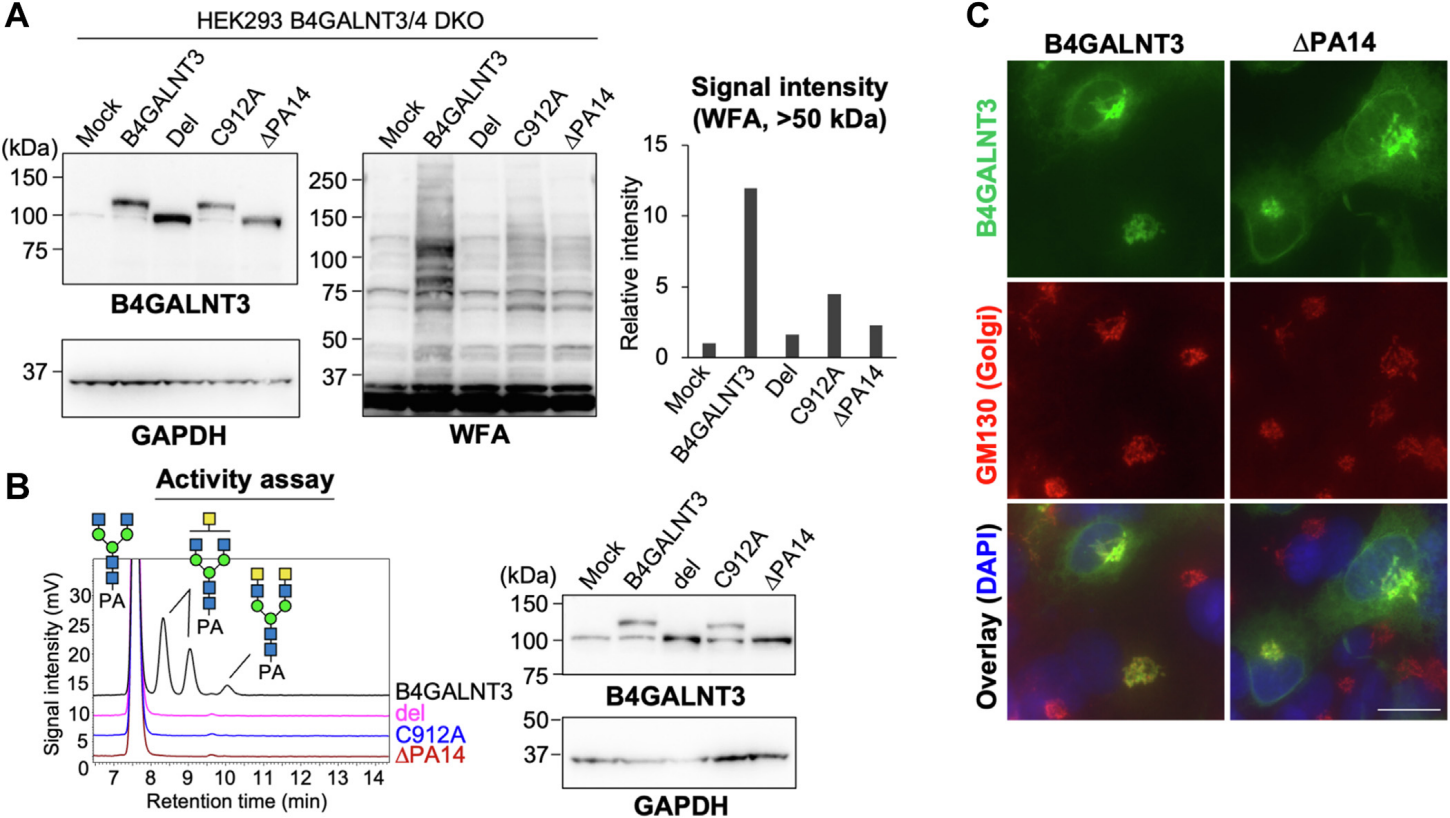
**Reduced activity of B4GALNT3 mutant lacking the PA14 domain for *N*-glycans.***A*, HEK293 DKO cells were transfected with the expression plasmid for B4GALNT3 WT, Del, C912A, ΔPA14, or the empty vector (Mock). The cell lysates were blotted with anti-B4GALNT3, anti-GAPDH, and WFA. The signal intensity of the bands (>50 kDa) blotted with WFA was quantified (*right graph*). *B*, lysates of HEK293 DKO cells expressing B4GALNT3 WT or mutants were incubated with GnGnbi-PA, and the reaction mixture was analyzed by reverse-phase HPLC (*left*). The lysates were subjected to Western blotting for B4GALNT3 and GAPDH (*right*). *C*, COS7 cells expressing B4GALNT3 WT or ΔPA14 were immunostained for B4GALNT3 (*green*) and the Golgi marker GM130 (*red*). Nuclei (*blue*) were counterstained with 4′,6-diamidino-2-phenylindole. Scale bar represents 20 μm. DKO, double knockout; HEK293, human embryonic kidney 293 cell line; WFA, *Wisteria floribunda* agglutinin.

Next, we investigated the activity of ΔPA14 mutant. Exogenous expression of ΔPA14 in HEK293 DKO cells brought about only a slight increase in WFA reactivity, compared with the robust increase of WFA reactivity by WT B4GALNT3 (Fig. 2*A*). C912A mutant also showed a decrease in WFA reactivity, suggesting that the disulfide bonding between PA14 domain and the C-terminal catalytic region is important for the activity. Moreover, the *in vitro* activity of ΔPA14 toward *N*-glycan substrate was almost negligible (Fig. 2*B*), and similar weak reactivity of WFA in ΔPA14-expressing cells was also observed in other cell lines, COS7 and Neuro2A (Fig. S1, *B* and *C*). Because the ΔPA14 mutant is mainly localized in the Golgi apparatus (Fig. 2*C*), similarly to the WT enzyme, it is suggested that the overall protein folding of the mutant is not abrogated and that this mutant is not entrapped in the endoplasmic reticulum. These findings collectively suggest that the PA14 domain is required for the enzyme activity of B4GALNT3.

### PA14-less B4GALNT3 is also inactive toward O-GalNAc-type substrates and glycoproteins

**Figure 3:**
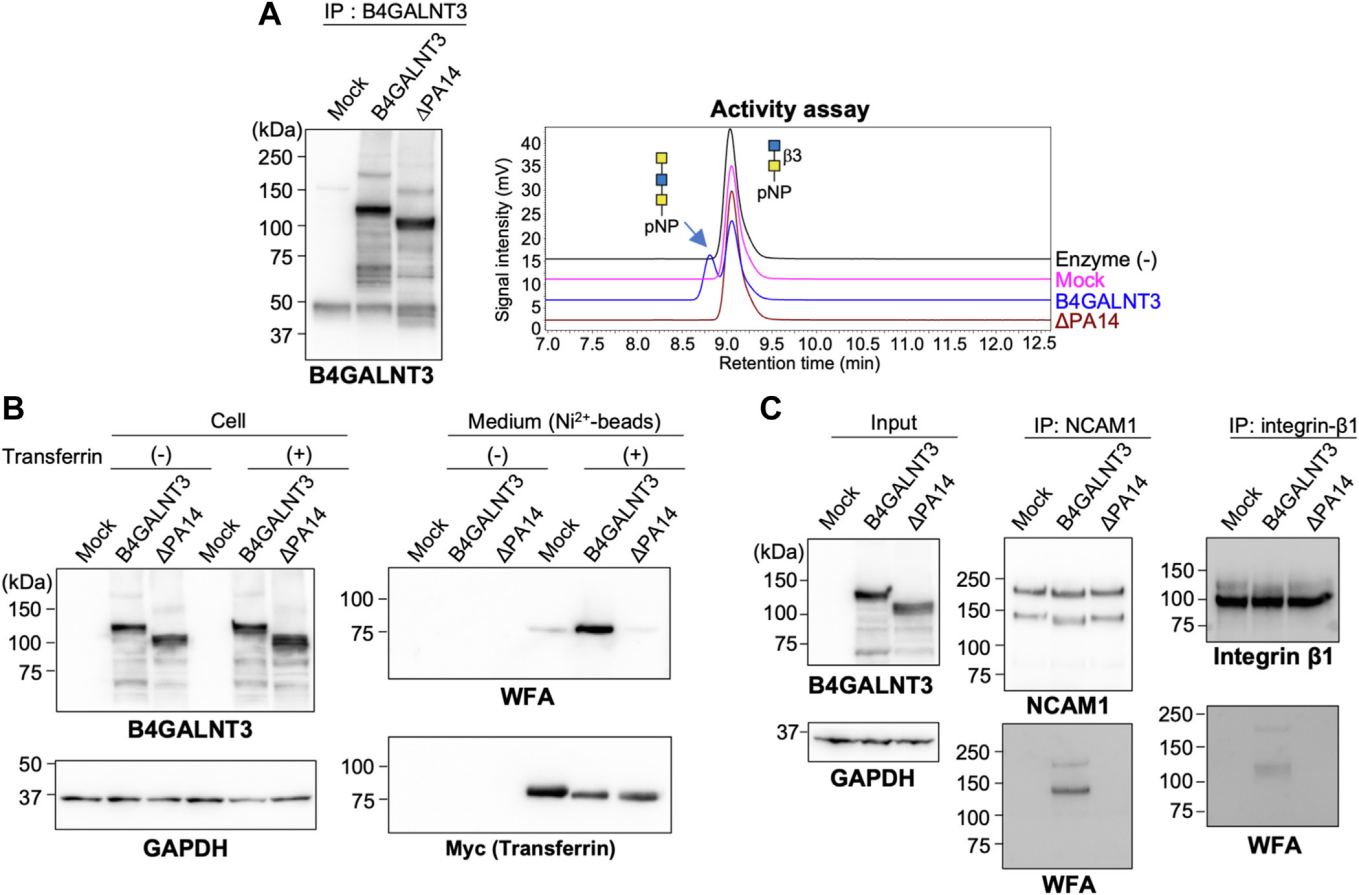
**Reduced activity of B4GALNT3 ΔPA14 for *O*-GalNAc glycan and glycoprotein.***A*, B4GALNT3 WT or ΔPA14 mutant was expressed in COS7 cells, immunoprecipitated, and blotted with anti-B4GALNT3 (*left*). The immunoprecipitated enzymes were incubated with GlcNAcβ1-3GalNAc-pNP, and the reaction mixture was analyzed by reverse-phase HPLC. *B*, COS7 cells were cotransfected with the expression plasmids for transferrin-mycHis and B4GALNT3 WT or ΔPA14. The cell lysates were subjected to Western blotting for B4GALNT3 and GAPDH (*left*). Secreted transferrin in the culture medium was purified using Ni^2+^ beads, and the proteins bound to the beads were blotted with WFA and anti-myc (*right*). *C*, Neuro2A cells were transfected with the expression plasmid for B4GALNT3 WT or ΔPA14 or an empty vector. The lysates (input) were subjected to immunoprecipitation with anti-NCAM1 or anti-integrin-β1. Proteins bound to the beads were blotted with anti-NCAM1, anti-integrin-β1, and WFA. WFA, *Wisteria floribunda* agglutinin.

The PA14 domain in yeast glucosidase was shown to determine the size of acceptor sugars, which are accommodated by the catalytic pocket (33). In addition, mammalian B4GALNT3 can act on *O*-GalNAc glycans, which are smaller than *N*-glycans (9, 18), as well as native glycoprotein substrates much larger than free *N*-linked oligosaccharides (17). On the basis of this background, we tested a smaller *O*-GalNAc-type substrate and larger glycoprotein substrates to examine the activity of B4GALNT3 WT and ΔPA14 mutant. While we detected the *in vitro* activity of immunopurified WT B4GALNT3 toward core 3 *O*-GalNAc disaccharide (GlcNAcβ1-3GalNAcα), activity of ΔPA14 was not observed (Fig. 3*A*), demonstrating that the PA14 domain is also required even for a smaller acceptor substrate.

To characterize the B4GALNT3 activity toward glycoproteins, we examined the increase in WFA reactivity of both exogenously and endogenously expressed glycoproteins. First, myc-His-tagged transferrin was coexpressed with B4GALNT3 in COS7 cells, and secreted transferrin was purified using Ni^2+^ beads from the culture medium. Lectin and Western blotting of purified transferrin showed a drastic increase in WFA reactivity by coexpression with B4GALNT3, but not with ΔPA14 (Fig. 3*B*), indicating that ΔPA14 is almost inactive toward transferrin. We also used Neuro2A cells and tested two endogenous glycoproteins, NCAM1 and integrin-β1, which are expressed in Neuro2A cells. The overexpression of B4GALNT3 resulted in an increase in WFA reactivity of both immunoprecipitated NCAM1 and integrin-β1, whereas that of ΔPA14 did not (Fig. 3*C*). Taken together, these findings demonstrated that ΔPA14 almost completely lost the activity toward several acceptor substrates, suggesting that the PA14 domain is essential for this activity and forms part of the catalytic region.

### N-Glycomics of B4GALNT3-expressing cells

**Figure 4:**
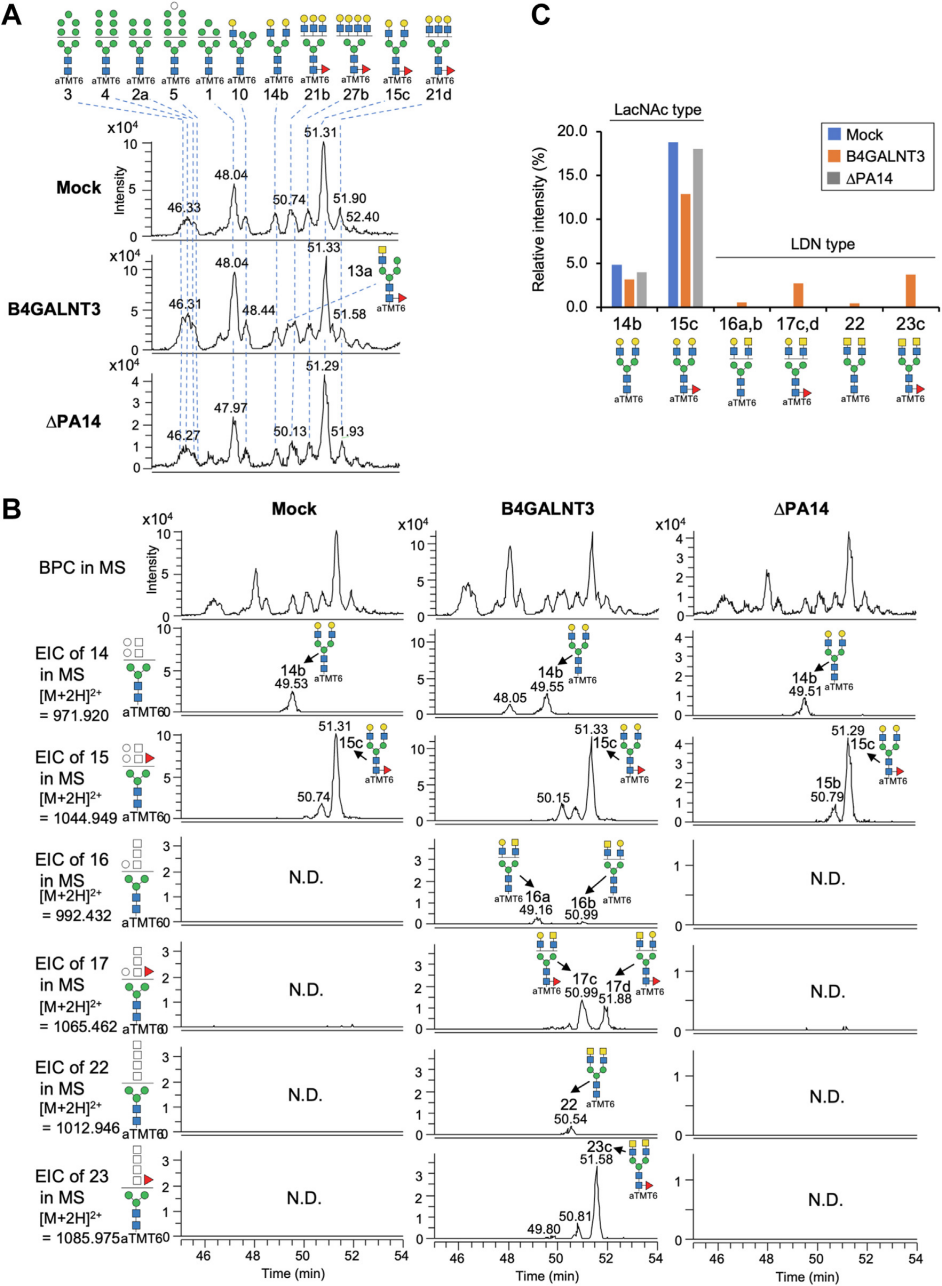
***N*-Glycomic analysis of B4GALNT3-expressing cells.***A*, base peak chromatograms (BPCs) from the LC–ESI–MS analysis of aTMT6-labeled desialylated *N*-glycans from Neuro2A cells transfected with the expression plasmid for B4GALNT3 WT or ΔPA14 or the empty vector (Mock) are shown. The deduced structures of the major *N*-glycans are depicted. *B*, extracted ion chromatograms (EICs) of the major biantennary *N*-glycans bearing LacNAc or LDN are shown. *C*, the signal intensities of biantennary complex-type glycans with terminal LacNAc or LDN in LC–MS analysis are shown. ESI, electrospray ionization; LDN, LacdiNAc.

To directly observe the *N*-glycan structural changes induced by B4GALNT3, we next performed *N*-glycomic analysis. B4GALNT3 WT or ΔPA14 was expressed in Neuro2A cells, and the cellular *N*-glycans were released, desialylated, labeled with aminoxy tandem mass tag sixplex (aTMT6), and analyzed by LC–electrospray ionization (ESI)–mass spectrometry (MS) (Fig. 4, *A* and *B*, S2 and Table. S1). As expected, we found a significant increase in LDN in cellular *N*-glycans by B4GALNT3 overexpression (Fig. 4, *B* and *C*). In contrast, ΔPA14 overexpression hardly increased LDN levels, consistent with the enzyme assays. Interestingly, we did not detect glycans bearing both bisecting GlcNAc and LDN, whereas a glycan potentially bearing bisecting GlcNAc and LacNAc was detected (Fig. S2), suggesting that LDN biosynthesis is negatively affected by bisecting GlcNAc.

### LDN biosynthesis by B4GALNT3 is inhibited by bisecting GlcNAc

**Figure 5:**
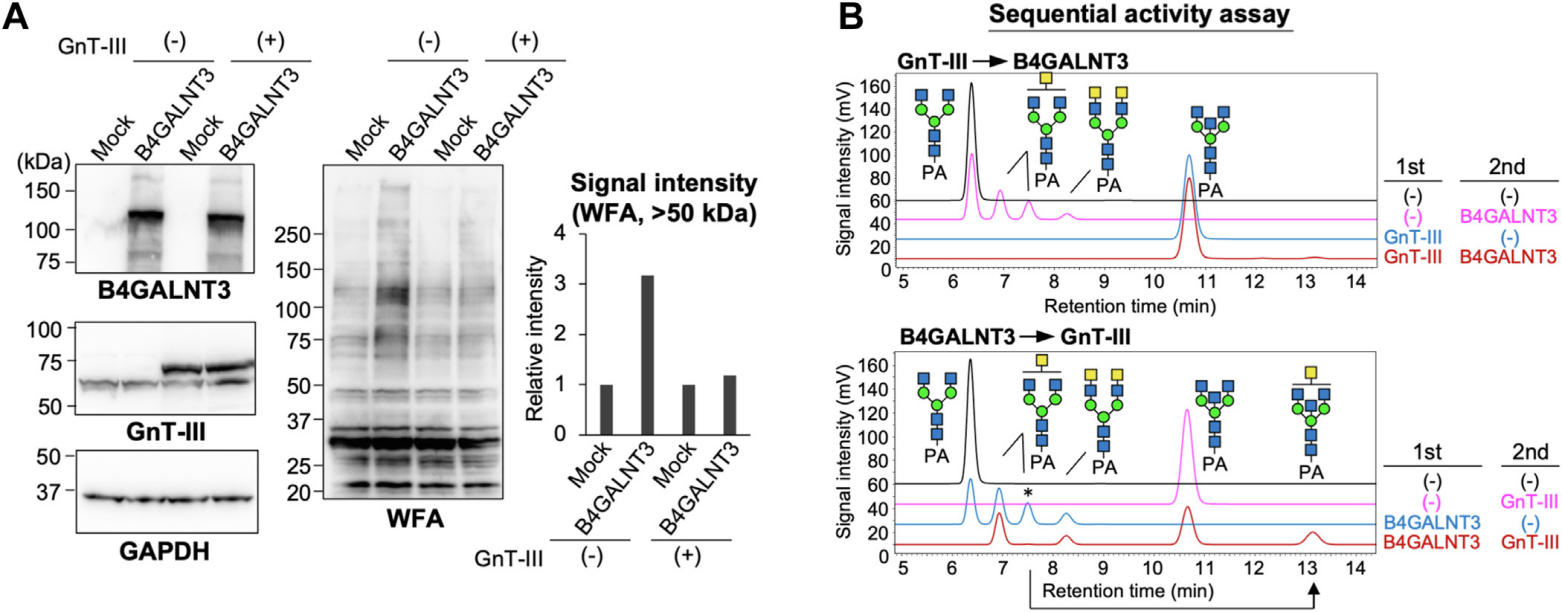
**Interplay of biosynthesis of LDN and bisecting GlcNAc.***A*, COS7 cells were transfected with the expression plasmid for B4GALNT3 with or without the plasmid for GnT-III. The cell lysates were blotted with anti-B4GALNT3, anti-GnT-III, anti-GAPDH, and WFA. The signal intensity of the bands (>50 kDa) blotted with WFA was quantified (*right graph*). *B* (*upper*), GnGnbi-PA was incubated with purified GnT-III or vehicle with UDP-GlcNAc. Purified B4GALNT3 or vehicle and UDP-GalNAc were added for the second step reaction. The reaction mixture was analyzed by reverse-phase HPLC. *Lower*, GnGnbi-PA was incubated with purified B4GALNT3 or vehicle with UDP-GalNAc. Purified GnT-III or vehicle and UDP-GlcNAc were added for the second step reaction. The reaction mixture was analyzed by reverse-phase HPLC. LDN, LacdiNAc; WFA, *Wisteria floribunda* agglutinin.

On the basis of the results of glycomic analysis, we next focused on the interplay between biosynthesis of LDN and bisecting GlcNAc. Bisecting GlcNAc is solely biosynthesized by GnT-III (MGAT3) (42) and was reported to suppress the activity of many downstream glycosyltransferases due to the conformational change of *N*-glycan (43, 44). Overexpression of GnT-III suppressed the formation of WFA-positive glycans by B4GALNT3 (Fig. 5*A*, right, second, and fourth lanes), suggesting that LDN biosynthesis is suppressed by the presence of bisecting GlcNAc. We also performed a sequential enzyme assay *in vitro* using purified soluble GnT-III and B4GALNT3 (Y83) (Fig. 5*B*). The results showed that the prior formation of bisecting GlcNAc by GnT-III inhibited the activity of B4GALNT3 (Fig. 5*B*, *upper, brown line*), consistent with the results of cellular experiments (Fig. 5*A*). Interestingly, inhibition in the opposite direction was partially observed, and prior action of B4GALNT3 inhibited GnT-III activity in a glycan-structure-dependent manner (Fig. 5*B*, *lower, brown line*). Among three B4GALNT3 products, only one glycan presumably bearing one GalNAc residue on either Man arm can be modified by GnT-III (Fig. 5*B*, *lower, arrow*). This strongly suggests that LDN inhibits GnT-III action in a branch-specific manner. These findings demonstrated that the biosyntheses of LDN and bisecting GlcNAc affect each other.

### LDN inhibits *N*-glycan capping

**Figure 6:**
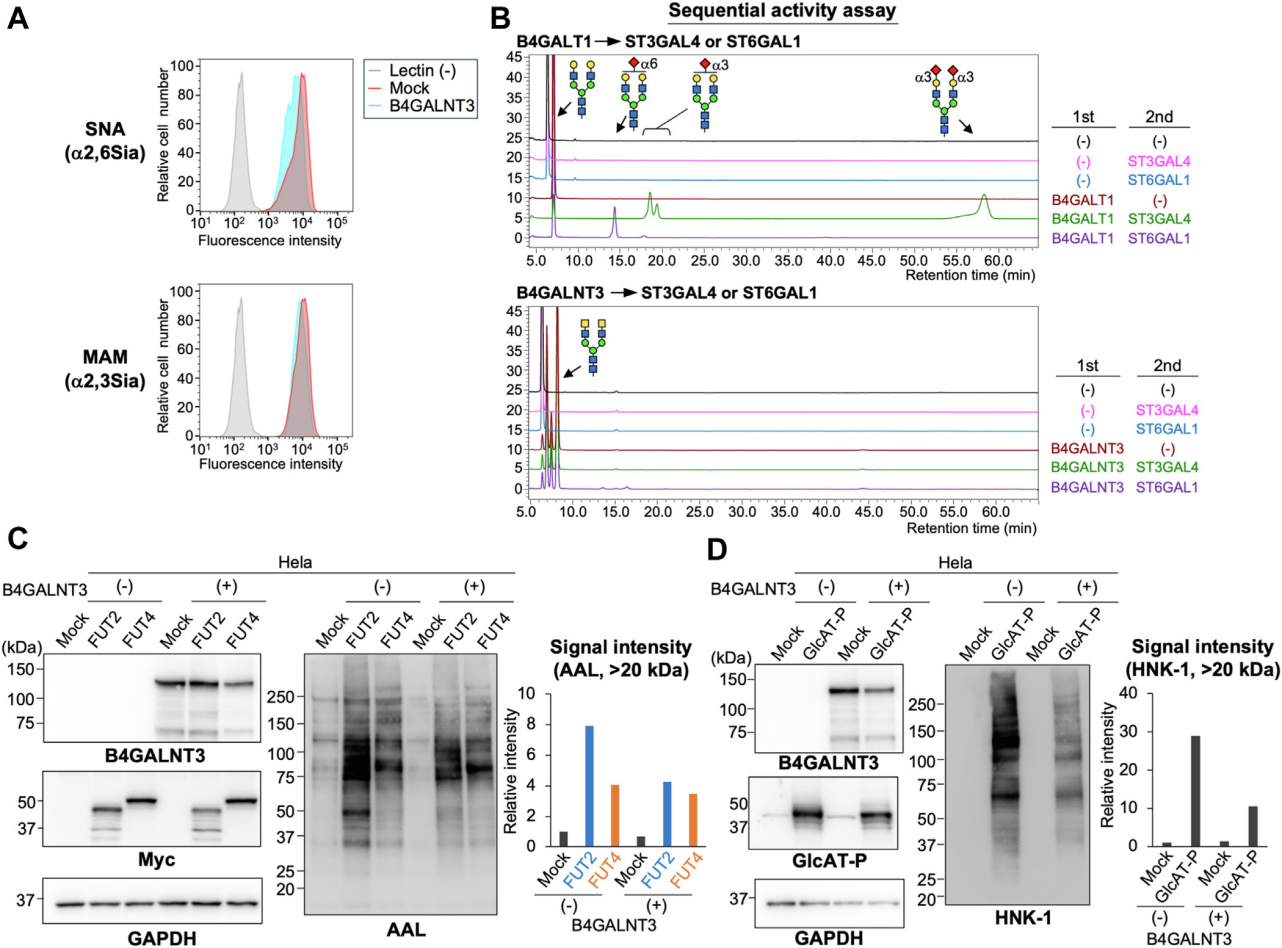
**Effects of LDN on terminal modifications of *N*-glycans.***A*, flow cytometry analysis of HEK293 DKO cells expressing B4GALNT3 with MAM and SNA lectins. B4GALNT3-expressing cells without lectin staining were used as a negative control (Lectin [−]). *B*, GnGnbi-PA was incubated with purified B4GALT1 or vehicle with UDP-Gal (u*pper*) or with purified B4GALNT3 or vehicle with UDP-GalNAc (*lower*). Then, ST3GALT4, ST6GAL1, or vehicle and CMP-NeuAc were added for the second step reaction. The reaction mixture was analyzed by reverse-phase HPLC. *C*, HeLa cells were transfected with the expression plasmid for FUT2-myc or FUT4-myc with or without the plasmid for B4GALNT3. The cell lysates were blotted with anti-B4GALNT3, anti-myc, anti-GAPDH, and AAL. The signal intensity of the bands (>20 kDa) blotted with AAL was quantified (*right graph*). *D*, HeLa cells were transfected with the expression plasmid for GlcAT-P and HNK-1ST with or without the plasmid for B4GALNT3. The cell lysates were blotted with anti-B4GALNT3, anti-GlcAT-P, anti-GAPDH, and HNK-1 mAb. The signal intensity of the bands (>20 kDa) blotted with HNK-1 mAb was quantified (*right graph*). AAL, *Aleuria aurantia* lectin; DKO, double KO; HEK293, human embryonic kidney 293 cell line; HNK, human natural killer; LDN, LacdiNAc; MAM, Ma*ackia amurensis* lectin; SNA, *Sambucus nigra* lectin.

Notably, the expression of B4GALNT3 caused a downshift of NCAM1 and integrin-β1 in the SDS-PAGE gel (Fig. 3*C*). Based on these results, we hypothesized that B4GALNT3 expression induced further changes in *N*-glycan structures. We thus examined the effects of LDN on the terminal modifications of *N*-glycans, such as sialylation, fucosylation, and HNK-1 biosynthesis. Consistent with a previous report showing that the removal of LDN by knocking out B4GALNT3 and 4 increased sialylation (36), overexpression of B4GALNT3 resulted in decreases in the reactivity of Sia-recognizing lectins, *Maackia amurensis* lectin (MAM) and *Sambucus nigra* lectin (SNA) (Fig. 6*A*). Furthermore, sequential *in vitro* activity assays using recombinant enzymes revealed that the major sialyltransferases for *N*-glycan synthesis, ST3GAL4 (45) and ST6GAL1 (46), showed lower activity toward LDN-containing *N*-glycan acceptor than toward LacNAc-containing glycan (Fig. 6*B*), demonstrating the negative effect of LDN on terminal sialylation.

We also examined whether fucosyltransferases acting on *N*-glycan terminal similarly had weaker activity toward LDN glycans than toward LacNAc-containing ones (Fig. 6*C*). B4GALNT3 was coexpressed with FUT2 or FUT4 that transfers Fuc to 2-OH of Gal for the biosynthesis of H-type antigen or to 3-OH of GlcNAc for the biosynthesis of Le^x^ antigen, respectively (47). The overexpression of either FUT2 or FUT4 resulted in an increase in reactivity to Fuc-recognizing *Aleuria aurantia* lectin (AAL) (Fig. 6*C*) in HeLa cells as shown in our previous study (43), and this reactivity was largely abolished by PNGaseF (Fig. S3*A*), indicating that the intracellular activity of these enzymes toward *N*-glycans can be evaluated by AAL reactivity. Because GalNAc residue has an N-acetyl group at the C2 position, FUT2 is probably unable to act on LDN. Indeed, as expected, B4GALNT3 expression weakened the enhancement of AAL reactivity by FUT2 overexpression (Fig. 6*C*, right, second, and fifth lanes), showing that LDN blocks FUT2 activity in cells. Meanwhile, the expression of B4GALNT3 barely decreased the levels of FUT4 products (Fig. 6*C*, right, third, and sixth lanes). This suggests that LDN does not affect the FUT4-mediated addition of Fuc to the inner GlcNAc residue, namely, biosynthesis of the Le^x^ epitope, and implies that not all terminal modifications are suppressed by LDN.

We also examined the biosynthesis of the HNK-1 epitope on LDN. HNK-1 is a brain-specific glycoepitope comprising HSO_3_–3GlcAβ1–3LacNAc and is involved in learning and memory (48). The rate-limiting enzyme for HNK-1 biosynthesis in the brain is a glucuronyltransferase, GlcAT-P (B3GAT1) (49), but it is unclear whether this enzyme can modify LDN as an acceptor. We confirmed that the exogenous expression of GlcAT-P led to emergence of the HNK-1 epitope on *N*-glycans of various glycoproteins in cells (Figs. 6*D* and S3*B*) (43). Interestingly, coexpression of GlcAT-P with B4GALNT3 largely inhibited the synthesis of the HNK-1 epitope (Fig. 6*D*). This demonstrates that LDN suppresses HNK-1 synthesis. Taking these findings together, the production of LDN by B4GALNT3 has significant negative impacts on the expression of the terminal modifications of *N*-glycans.

## Discussion

**Figure 7:**
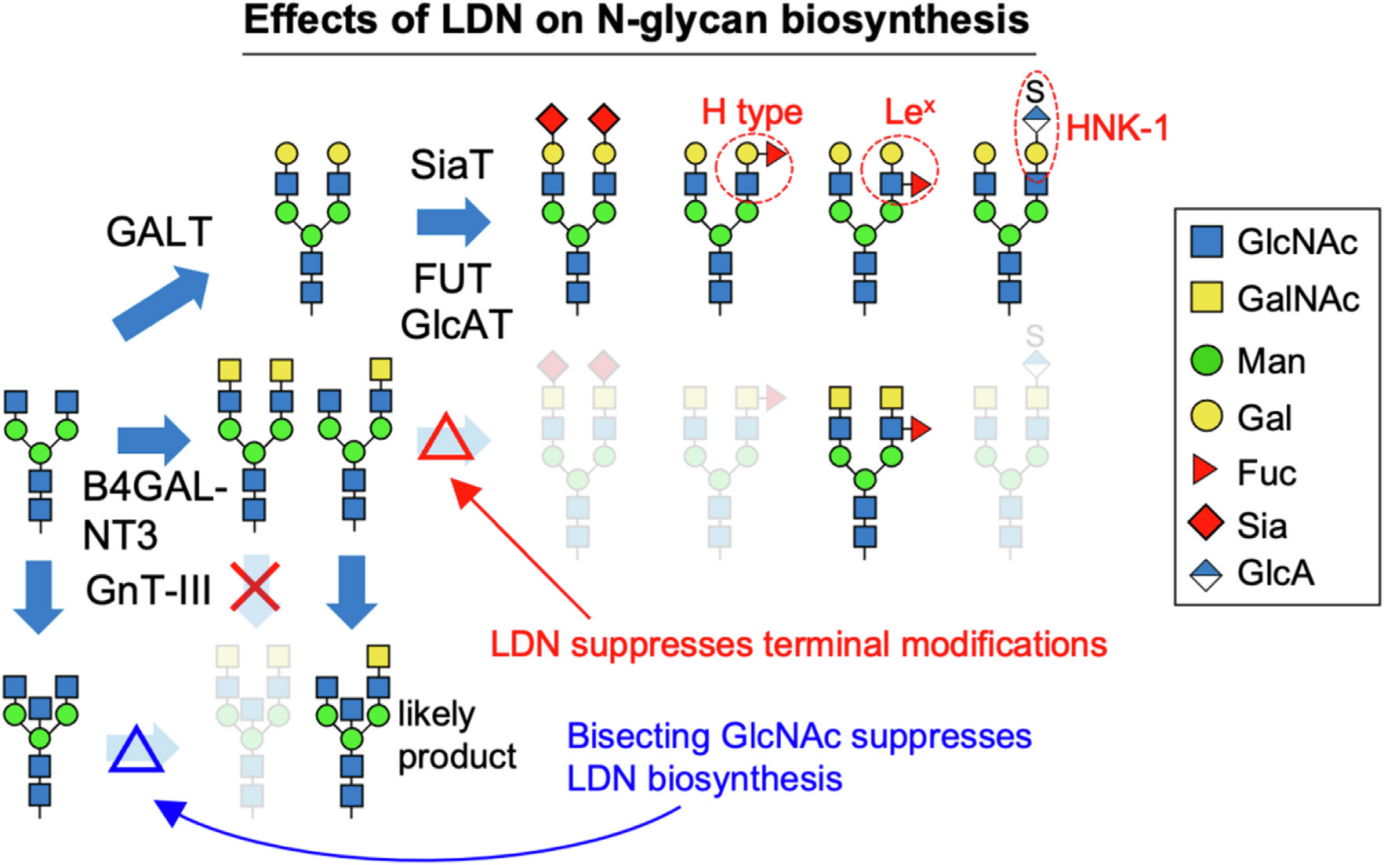
**Suggested pathway for biosynthesis of LDN- and LacNAc-containing *N*-glycans.** LDN, LacdiNAc.

In this study, we revealed that the LDN synthase B4GALNT3 has a unique PA14 domain that is essential for the enzyme activity. We also discovered new relationships between LDN and other glycan epitopes, which greatly impact the *N*-glycan profile. In detail, the biosyntheses of LDN and bisecting GlcNAc are mutually suppressive. In addition, terminal sialylation, HNK-1 synthesis, and α1,2-fucosylation were found to be inhibited by LDN expression (Fig. 7). These findings underscore the importance of LDN in *N*-glycan maturation as well as in regulation of the glycoprotein half-life in circulation, as reported previously.

The PA14 domain is only found in the B4GALNTs among mammalian glycosyltransferases. This domain was shown to interact with glycans (34, 35). In the case of glycohydrolases, the PA14 domain was shown to determine the chain length of acceptable substrate glycans. *Kluyveromyces marxianus Km*BglI and *Hordeum vulgare* ExoI are structurally similar glucosidases (33), but only *Km*BglI has a PA14 domain. *Km*BglI is strictly specific to disaccharides, whereas ExoI prefers longer oligosaccharides. In the same GH3 group in the CAZy database, PA14 domain–containing glycosidases (*At*CbgI and *Vv*BglII) are all specific to small substrates (33). In these enzymes, the PA14 domain covers an active site to form a narrow catalytic pocket and to accept only small sugars. In yeast adhesin cea1, the PA14 domain interacts with GlcNAc (Fig. S4*B*) (35). In the case of B4GALNT3, our model predicted that the putative glycan-binding site of the PA14 domain is close to the donor substrate–binding site (Fig. S4). This suggests that the PA14 domain in B4GALNT3 is located close to the catalytic pocket and possibly acts in the same way as *Km*BglI. Alternatively, there is another possibility that the independent recognition of ligand glycan by PA14 might trigger the action of the catalytic domain. We recently reported that several mammalian glycosyltransferases, such as GnT-V and GnT-IVa have their own unique noncatalytic domains in the luminal region to regulate enzymatic activity and substrate specificity (25, 26, 50), implying that the PA14 domain of the B4GALNTs also plays important roles in activity or specificity. To further clarify the role of the PA14 domain in B4GALNT3 action, there is a need to obtain the 3D structure of the whole luminal region in complex with substrates.

Previous studies showed that B4GALNT3 recognizes the specific polypeptide motifs (17, 18, 19), which was suggested to be a mechanism for protein-selective action of B4GALNT3. Although our present work has a limitation of using overexpression system, our data suggested that B4GALNT3 has a potential to modify various kinds of glycoproteins, including transferrin, NCAM1, and integrin-β1, under these conditions. This raised a possibility that B4GALNT3 can accept a wider range of substrate proteins than previously thought.

Regarding the functions of LDN, *B4galnt3*-KO mice showed an aberrantly high level of sclerostin in blood, concomitant with abnormal bone loss (16). Given the negative effects of LDN on sialylation, we consider that the longer high-life and increased level of sclerostin in *B4glant3*-KO blood are probably caused by increased sialylation of sclerostin. Furthermore, recombinant coagulation factor VII produced from B4GALNT3 and 4 DKO cells showed increased sialylation, weaker binding to asialoglycoprotein receptor and mannose receptor, and a longer half-life in blood (36). On the basis of these findings, we hypothesized that one of the most important physiological functions of LDN is to suppress terminal modifications of *N*-glycans on secreted proteins to control their levels in circulation.

We showed that the presence of LDN in acceptor *N*-glycans affects the actions of various glycosyltransferases. Regarding GnT-III, only one LDN product out of three can be accepted as a substrate (Fig. 5*B*, *lower*). In the case of LacNAc, the presence of Gal on the α1,3-arm was already demonstrated to almost completely prevent GnT-III action, whereas Gal on the α1,6-arm is tolerated by GnT-III (51). Although the 3D structure and the mechanism of substrate recognition of GnT-III have not been clarified, these findings suggest that the presence of LDN on the α1,3-Man arm also inhibits the action of GnT-III. The intra-Golgi localization and reaction orders of glycosyltransferases are critically important for the biosynthesis of final glycan products. As GnT-III and B4GALNT3 were previously suggested to be localized in medial- and trans-Golgi, respectively (52, 53), it is more likely that GnT-III precludes the action of B4GALNT3 in cells.

Regarding sialyltransferases, fucosyltransferases, and HNK-1 enzymes, we also found that the actions of ST3GAL4, ST6GAL1, FUT2, and GlcAT-P were suppressed by LDN, whereas that of FUT4 was not inhibited by LDN. To structurally examine whether LDN can fit the substrate binding pockets of these enzymes, we generated docking models of FUT4 and GlcAT-P with LDN, using crystal structures of GlcAT-P and FUT4 homolog FUT9 (Fig. S5). In FUT4-LDN model, *N*-acetyl group at C2 position of GalNAc is exposed and seems to interfere with neither the enzyme nor the donor substrate (Fig. S5*A*). In contrast, in GlcAT-P-LDN model, the *N*-acetyl group of GalNAc would be too close to the donor sugar, likely causing a steric hindrance (Fig. S5*B*). These findings are well consistent with the biochemical results (Fig. 6, *C* and *D*) and further suggest that LDN can affect the activity of enzymes acting on *N*-glycan termini.

In conclusion, we here revealed the unique domain organization of B4GALNT3 and the important roles of this enzyme in *N*-glycan maturation. To further understand the mechanism by which the PA14 domain is involved in enzymatic reactions, it will be necessary to solve the 3D structure of B4GALNT3 in complex with substrates. This could also help us understand the selectivity of B4GALNTs for glycoproteins as acceptors. In addition, the effects of LDN on the biosynthesis of other glycoepitopes and *O*-GalNAc glycans that were not tested in this study should be explored. Clarifying these issues in the future would provide us with new clues to elucidate how the complex system of glycan biosynthesis is organized and regulated in mammalian cells.

## Experimental procedures

### Reagents

The following antibodies and lectins were used: mouse anti-GAPDH (Merck Millipore; MAB374), mouse anti-Myc (Millipore; 05-724), rabbit anti-B4GALNT3 (HPA011404, for Western blotting, immunoprecipitation, and immunostaining), rabbit anti-B4GALNT3 (Novus Biologicals; NBP-2-84488, for Western blotting in Figs 1*E* and 2*B*), rabbit anti-NCAM (Abcam; ab95153), goat anti-integrin-β1 (R&D; AF2405), rabbit anti-MGAT3 (Proteintech; 17869-1-AP), HNK-1 mAb (ATCC; clone Leu7), mouse anti-GM130 (BD Biosciences; 610822), horseradish peroxidase (HRP)-anti-mouse IgG (GE Healthcare; NA931V), HRP-anti-goat IgG (Jackson ImmunoResearch; 705-035-147), HRP-anti-rabbit IgG (GE Healthcare; NA934V), HRP-anti-mouse IgM (Invitrogen; 62-6802), Alexa546-anti-mouse IgG (Invitrogen; A10036), Alexa488-anti-rabbit IgG (Invitrogen; A21206), biotinylated WFA (Sigma; L1516), biotinylated AAL (Vector Laboratories; B-1395), FITC-SNA (Vector Laboratories; FL-1301), and FITC-MAM (Seikagaku Corporation). Rabbit anti-GlcAT-P (GP2) was provided by Dr Shogo Oka (Kyoto University). GlcNAcβ1-3GalNAcα-pNP was purchased from Tokyo Chemical Industry, Co.

### Plasmid construction

Primers for plasmid construction are listed in Table S2. Complementary DNA (cDNA) of full-length human B4GALNT3 was amplified by PCR using reverse-transcribed total RNA from NTERA cells and cloned into EcoRI–EcoRV sites of pcDNA6-mycHisA by Gibson assembly to construct pcDNA6-mycHisA/B4GALNT3. To construct a plasmid for expressing B4GALNT3 lacking the PA14 domain (pcDNA6-mycHisA/B4GALNT3 Del), the two fragments were amplified by PCR, and the resultant fragments were inserted into EcoRI–EcoRV sites of pcDNA6-mycHis A by Gibson assembly. As a result, PA14 domain was replaced with a linker sequence (GGGSGS). Point mutation (C912A) was introduced by PCR with pcDNA6-mycHisA/B4GALNT3 or pcDNA6-mycHisA/B4GALNT3 Del as a template using QuikChange Lightning Site-Directed Mutagenesis Kit. To construct plasmids for soluble B4GALNT3 (S60, Y83, and S100), the DNA fragments were amplified by PCR using pcDNA6-mycHisA/B4GALNT3 as a template, and the fragments were inserted into EcoRI–EcoRV sites of pcDNA-IH (54). Human transferrin cDNA was amplified by PCR using the human liver cDNA library (TAKARA; Human MTC Panel I) and cloned into EcoRI–XhoI sites of pcDNA6-mycHisA by Gibson assembly. The construction of pcDNA6-mycHisA/human GnT-III, pcDNA-IH/human GnT-III, proteinA-mouse B4GALT1, pcDNA-IH/human ST3GAL4, pcDNA-IH/human ST6GAL1, pcDNA6-mycHisA/mouse FUT2, and pcDNA6-mycHisA/mouse FUT4 was previously described (43). pIRES/rat GlcAT-P + rat HNK-1ST was constructed as described previously (55).

### Cell culture

HEK293 (ATCC), HEK293 B4GALNT3/4 DKO, COS7 (RIKEN Cell Bank), Neuro2A (RIKEN Cell Bank), and HeLa (RIKEN Cell Bank) cells were grown at 37 °C under 5% CO_2_ conditions in Dulbecco's modified Eagle's medium supplemented with 10% fetal bovine serum and 50 μg/ml kanamycin. HEK293 B4GALNT3/4 DKO cells were kindly provided by Dr Morihisa Fujita (Gifu University) (7).

### Plasmid transfection

Cells cultured on a 10-cm or 6-cm dish were transfected with 5 μg or 2 μg of plasmid using Lipofectamine 3000 Transfection Reagent (Thermo Fisher Scientific), in accordance with the manufacturer’s protocol. For the expression of recombinant soluble B4GALNT3, 15 μg of the plasmid was transfected into COS7 cells cultured on a 15-cm dish using Polyethyleneimine MAX (Polyscience).

### Structural representation

The putative 3D structures of human B4GALNT3 (UniProtKB: Q9L9W6) and human FUT4 (UniProtKB: P22083) were generated by AlphaFold2 (56). Structural superpositions of Cea1 (Protein Data Bank [PDB] ID: 5A3M) or DmB4GalT (PDB ID: 4LW3) onto human B4GALNT3 as well as human FUT9 (PDB ID: 8D0Q) onto human FUT4 were performed using SUPERPOSE (57). Atomic structures of LDN and UDP-GlcA were extracted from human galectin-3 in complex with LDN (PDB ID: 7BE3) and GlcAT-I in complex with UDP-GlcA (PDB code: 1KWS), respectively. Structural superpositions of LDN onto LacNAc moiety in FUT9 complex and LacNAc in GlcAT-P complex and UDP-GlcA onto UDP moiety in GlcAT-P complex were performed with program LSQKAB (58). All structural figures were prepared with PyMOL (The PyMOL Molecular Graphics System, version 1.2r3pre; Schrödinger, LLC).

### Western and lectin blotting and Coomassie brilliant blue staining

Cells were sonicated in lysis buffer (Tris-buffered saline [TBS] containing 0.5% Nonidet P-40 [NP-40] and protease inhibitor cocktail [Fujifilm]). Protein concentrations of the lysates were measured using Pierce BCA Protein Assay (Thermo Fisher Scientific). The cell lysates, purified proteins, or immunoprecipitates were mixed with Laemmli sample buffer and subjected to 5 to 20% SDS-PAGE and Western and lectin blotting. For Coomassie brilliant blue staining, SDS-PAGE gel was stained with GelCode Blue Safe Protein Stain (Thermo Fisher Scientific). For Western blotting, proteins separated by SDS-PAGE were transferred to a nitrocellulose membrane. After blocking with TBS containing 5% skim milk and 0.1% Tween-20, the membranes were incubated with the primary antibodies overnight at 4 °C. After washing with TBS containing 0.1% Tween-20 (TBS-T), the membranes were incubated with the HRP-conjugated secondary antibodies at room temperature. After washing with TBS-T and TBS, signals were detected with Western Lightning Plus-ECL (PerkinElmer) or SuperSignal West Femto Maximum Sensitivity substrate (Thermo Fisher Scientific). For lectin blotting, nitrocellulose membranes were blocked with TBS-T, followed by incubation with biotinylated lectins overnight at 4 °C. After washing with TBS-T, the membranes were incubated with HRP–streptavidin (VECTASTAIN ABC Standard Kit). After washing with TBS-T and TBS, the protein bands were detected, the same as in the Western blotting. Images were taken using FUSION-SOLO 7s EDGE (Vilber-Lourmat), and the signals were quantified using ImageJ (National Institutes of Health). All the blotting experiments were independently performed multiple times to confirm reproducibility.

### Purification of recombinant proteins

The purification of recombinant soluble enzymes was carried out as described previously (43, 59). In brief, 60 to 80% confluent COS7 or HEK293T cells on 15-cm dishes were transfected with the plasmids using polyethylenimine MAX. After 6 h of transfection, the culture medium was replaced with Opti-MEM I, followed by further incubation for 72 h at 37 °C. The culture medium was collected, and the cell debris was removed by centrifugation. Then, 6× His-tagged enzymes were purified using a Ni^2+^ column and then desalted using a NAP-5 gel filtration column (Cytiva). Recombinant soluble 6× His-tagged human GnT-III, human ST3GAL4, and human ST6GAL1, and proteinA-tagged mouse B4GALT1 were prepared as described previously (43, 60).

### B4GALNT3 activity assay

To measure the activity toward *N*-glycans, cell lysate expressing B4GALNT3 or purified B4GALNT3 was incubated in 10 μl of reaction buffer (50 mM Tris–HCl, pH 7.4, 1 mM MnCl_2_, and 0.5% Triton X-100) containing 1 mM UDP-GalNAc and 10 μM fluorescently labeled acceptor substrate (GnGnbi-PA) at 37 °C for 3 h. The reaction mixture was boiled at 95 °C for 5 min to stop the reaction, followed by the addition of 40 μl of water. The mixture was centrifuged at 21,500*g* for 5 min, and 10 μl of the supernatant was injected into an HPLC system equipped with an ODS column (Inertsil ODS-3, 4.6 × 250 mm; GL Sciences) to detect the fluorescence-conjugated acceptor substrate and product. The mobile phase consisted of 80% buffer A (20 mM acetate buffer, pH 4.0 adjusted by aqueous ammonia) and 20% buffer B (buffer A containing 1% 1-butanol).

To measure the activity toward the *O*-GalNAc-type disaccharide (GlcNAcβ1-3GalNAcα-pNP), B4GALNT3 or its ΔPA14 mutant was expressed in COS7 cells and immunoprecipitated as described below. The enzyme-bound beads were incubated in 10 μl of reaction buffer (50 mM Tris–HCl, pH 7.4, 1 mM MnCl_2_, 0.5% Triton X-100) containing 1 mM UDP-GalNAc and 250 μM GlcNAcβ1-3GalNAcα-pNP at 37 °C for 16 h. The reaction mixture was boiled, diluted with water, and centrifuged in a similar way as for the *N*-glycan substrate. A total of 10 μl of the supernatant was injected into the HPLC system equipped with an ODS column to detect the acceptor substrate and the product. The mobile phase was 85% solvent A (20 mM acetate buffer, pH 4.0 adjusted by aqueous ammonia) and 15% solvent B (acetonitrile), and absorbance at 305 nm was monitored.

### Sequential activity assay

The sequential assays of GnT-III and B4GALNT3 were conducted under two sets of conditions. (i) Purified GnT-III was first incubated in 10 μl of reaction buffer (50 mM Tris–HCl, pH 7.4, 1 mM MnCl_2_, and 0.5% Triton X-100) containing 20 mM UDP-GlcNAc and 10 μM GnGnbi-PA at 37 °C for 30 min. Then, 6 μl of the reaction mixture was mixed with 3 μl of purified B4GALNT3 and 1 μl of 10 mM UDP-GalNAc, followed by incubation at 37 °C for 3 h. (ii) Purified B4GALNT3 was first incubated in 10 μl of reaction buffer (50 mM Tris–HCl, pH 7.4, 1 mM MnCl_2_, and 0.5% Triton X-100) containing 1 mM UDP-GalNAc and 10 μM GnGnbi-PA at 37 °C for 3 h. Then, 6 μl of the reaction mixture was mixed with 3 μl of purified GnT-III and 1 μl of 200 mM UDP-GlcNAc, followed by incubation at 37 °C for 3 h.

For the assays of B4GALT1 or B4GALNT3 and sialyltransferase, B4GALT1 or B4GALNT3 was first incubated in 12 μl of reaction buffer (125 mM Tris–HCl, pH 7.4, 2.5 mM MnCl_2_, and 1.25% Triton X-100) containing 1 mM of appropriate donor substrate (UDP-Gal or UDP-GalNAc) and 10 μM GnGnbi-PA at 37 °C for 6 h. B4GALT1 or B4GALNT3 was added to the mixture, followed by incubation for 16 h at 37 °C, and again the same enzyme was added to the mixture, followed by a further 18 h of incubation. Then, purified ST3GAL4 or ST6GAL1 was added to the mixture together with CMP-NeuAc (final concentration of 3.3 mM), followed by 3 h of incubation at 37 °C.

The reaction mixtures were boiled for 5 min, diluted with water, and centrifuged at 21,500*g* for 5 min. A total of 10 μl of the supernatant was injected into the HPLC system equipped with an ODS column. The mobile phase consisted of 80% buffer A (20 mM acetate buffer, pH 4.0 adjusted by aqueous ammonia) and 20% buffer B (buffer A containing 1% 1-butanol).

### PNGaseF treatment

For PNGaseF treatment, samples were denatured in denaturing buffer (TBS containing 1% NP-40, 0.5% SDS, 1% β-mercaptoethanol, and protease inhibitor mixture) at 95 °C for 5 min, followed by 10-fold dilution with PBS containing NP-40 (final concentration of 0.75% v/v). Samples were then incubated with water or PNGaseF (NEB; P0704) at 37 °C for 2 h. The samples were then mixed with 5× Laemmli sample buffer and incubated at 95 °C for 5 min.

### Immunofluorescence staining

Cells on an eight-well glass chamber slide were fixed with 4% paraformaldehyde/PBS at room temperature for 15 min, washed with PBS three times, and then permeabilized with 0.1% Triton X-100/1% bovine serum albumin/PBS at room temperature for 15 min. After washing the cells with PBS three times, they were incubated with primary antibodies for 60 min and then washed with PBS. Next, Alexa488- or Alexa546-conjugated secondary antibodies and 4′,6-diamidino-2-phenylindole were added to the cells, followed by further incubation for 30 min. After washing with PBS and removing the chamber, cells were mounted with mounting solution (ProLong Diamond Antifade Mountant; Invitrogen). Fluorescence was imaged using a BZ-X800 all-in-one fluorescence microscope (KEYENCE).

### Immunoprecipitation

Cells were suspended in lysis buffer (TBS containing 1% NP-40 and protease inhibitor mixture) and lysed by sonication. The cell lysates were centrifuged at 18,800*g* for 10 min, and the obtained supernatants were collected. Each collected supernatant was incubated with an antibody and Dynabeads protein G at 4 °C for 2 h or overnight. The beads were washed three times with 0.1% NP-40/TBS and then boiled with 1× Laemmli sample buffer at 95 °C for 5 min to elute the proteins. The samples were analyzed by SDS-PAGE and Western blotting. For the assay of activity toward the disaccharide (GlcNAcβ1-3GalNAcα-pNP), the enzyme-bound beads were directly used as an enzyme source.

### Preparation of secreted proteins from culture medium

After 4 h of transfection, the culture medium was replaced with Opti-MEM I, followed by a further 48 h of culture. The medium was collected and centrifuged at 1200*g* for 5 min to remove the cell debris, and the supernatants were incubated with Ni-Sepharose 6 Fast Flow (Cytiva), which had been preequilibrated with PBS. After 2 h of incubation, the beads were collected, washed with buffer (10 mM phosphate buffer [pH 7.4], 0.5 M NaCl, and 20 mM imidazole), and the bound proteins were eluted with elution buffer (10 mM phosphate buffer [pH 7.4], 0.5 M NaCl, and 0.5 M imidazole).

### *N*-Glycomics

*N*-Glycans from cell membrane proteins were released (61), labeled with aTMT6 reagent (Thermo Fisher Scientific), and finally analyzed by LC–ESI–MS, in accordance with the previously reported procedures with slight modifications (43, 50). Briefly, mouse Neuro2A cells (1 × 10^7^ cells each) were centrifuged after homogenization to remove nuclei and unbroken cells. The supernatant was ultracentrifuged, and then the membrane pellet was mixed with ice-cold acetone after purification by phase partitioning. After centrifugation, the precipitated membrane proteins were dissolved and spotted onto polyvinylidene difluoride (PVDF) membrane. After staining, the protein spots were excised from the PVDF membrane and placed into one of the wells of a 96-well plate. PNGaseF (Roche) was added to the spots after blocking of PVDF membrane. The released *N*-glycans were reacted with aTMT6 after desialylation with 2 M acetic acid (80 °C, 2 h). *N*-Glycans labeled with aTMT6 were separated on a carbon column (5 μm HyperCarb, 1 mm I.D. × 100 mm; Thermo Fisher Scientific), and the eluate was introduced continuously into an ESI source (LTQ Orbitrap XL; Thermo Fisher Scientific). MS spectra were obtained in positive ion mode using Orbitrap. For MS/MS analysis, the top three precursor ions were fragmented by high-energy collisional dissociation using stepped-collision energy *via* an Orbitrap. Monoisotopic masses were assigned possible monosaccharide compositions using a GlycoMod software tool (mass tolerance for precursor ions as [M + H]^+^ is ±0.01 Da, https://web.expasy.org/glycomod/), and the proposed glycan structures were further verified through annotation using a fragmentation mass-matching approach based on the MS/MS data. Xcalibur software, version 2.2 (Thermo Fisher Scientific) was used to show the base peak chromatogram and the extracted ion chromatogram, and to analyze the MS and MS/MS data. The relative abundances (%) of each glycan structure were calculated by setting the sum of peak intensities of all detected *N*-glycans labeled with aTMT6 in each extracted ion chromatogram as 100%.

### Fluorescence-activated cell sorting

Cells were washed with PBS and collected using cell scrapers, followed by centrifugation at 500*g* for 3 min. The cells were washed with fluorescence-activated cell sorting (FACS) buffer (1% bovine serum albumin/PBS) once and stained with FITC-labeled lectin in FACS buffer on ice for 30 min. Data were collected with a FACS Melody cell sorter and analyzed using FlowJo software (BD Biosciences).

## Data availability

Glycomic raw data for glycan structure analysis by LC–ESI–MS have been deposited in GlycoPOST (announced ID: GPST000412, URL: https://glycopost.glycosmos.org/entry/GPST000412) .All other data are contained within the article.

## Supporting information

This article contains supporting information.

## Conflict of interest

The authors declare that they have no conflicts of interest with the contents of this article.
