# Supplementary material for: LacdiNAc synthase B4GALNT3 has a unique PA14 domain and suppresses N-glycan capping
Source: J Biol Chem. 2024 Jun 4;300(7):107450. doi: 10.1016/j.jbc.2024.107450 (PMC11254600; doi:10.1016/j.jbc.2024.107450)
Supplement: Supplemental Figures S1–S5 and Table S2 [file mmc2.pdf]

Supporting Information

LacdiNAc synthase B4GALNT3 has a unique PA14 domain and suppresses *N*-glycan capping

Yuko Tokoro, Masamichi Nagae, Miyako Nakano, Anne Harduin-Lepers, Yasuhiko Kizuka*

*Correspondence: Yasuhiko Kizuka, Ph.D., kizuka.yasuhiko.k8@f.gifu-u.ac.jp

This Supporting information includes:

Figs. S1–S5 (included in this PDF)

Table S1-S2 (S1 is included in this PDF. S2 is separate file)

Figure S1

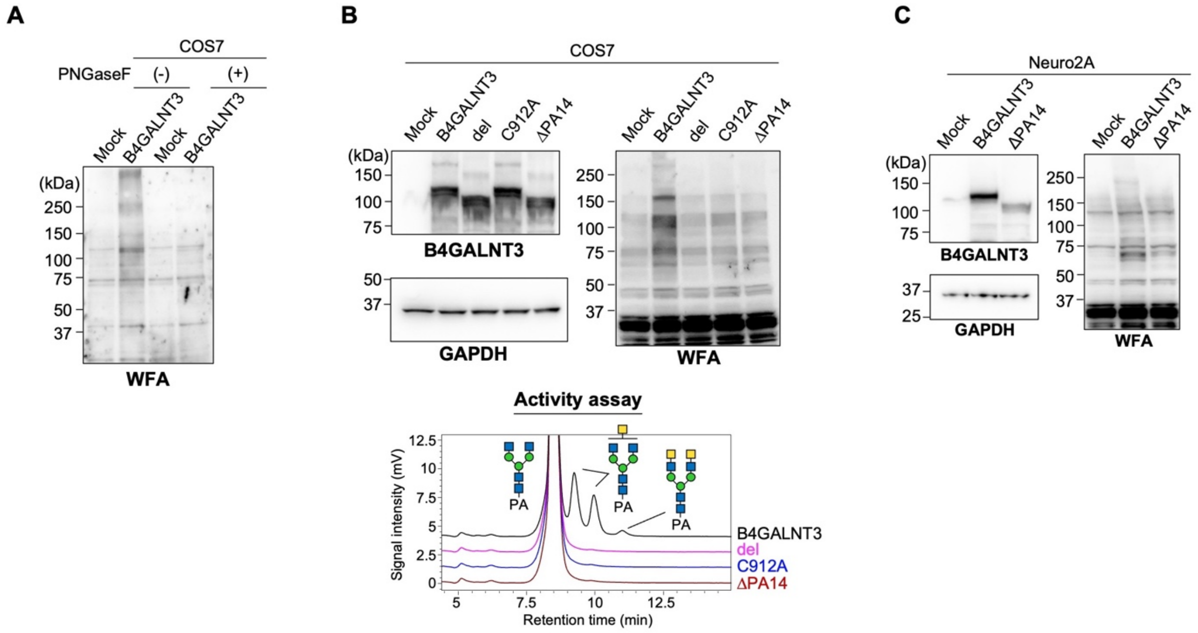

Figure S1. Activity of LDN synthase, B4GALNT3, in cells and *in vitro* enzyme assay. ***A***, COS7 cells were transfected with the plasmid for B4GALNT3, and the lysates were incubated with PNGaseF and analyzed by blotting with WFA lectin. ***B***, COS7 cells were transfected with the expression plasmid for B4GALNT3 WT, del, C912A, ΔPA14, or the empty vector (Mock). The cell lysates were blotted with anti-B4GALNT3, anti-GAPDH, and WFA (upper), or incubated with GnGnbi-PA, and the reaction mixture was analyzed by reverse-phase HPLC (lower). ***C***, Neuro2A cells were transfected with the expression plasmid for B4GALNT3 or the empty vector (Mock). The cell lysates were blotted with anti-B4GALNT3, anti-GAPDH, and WFA.

Figure S2

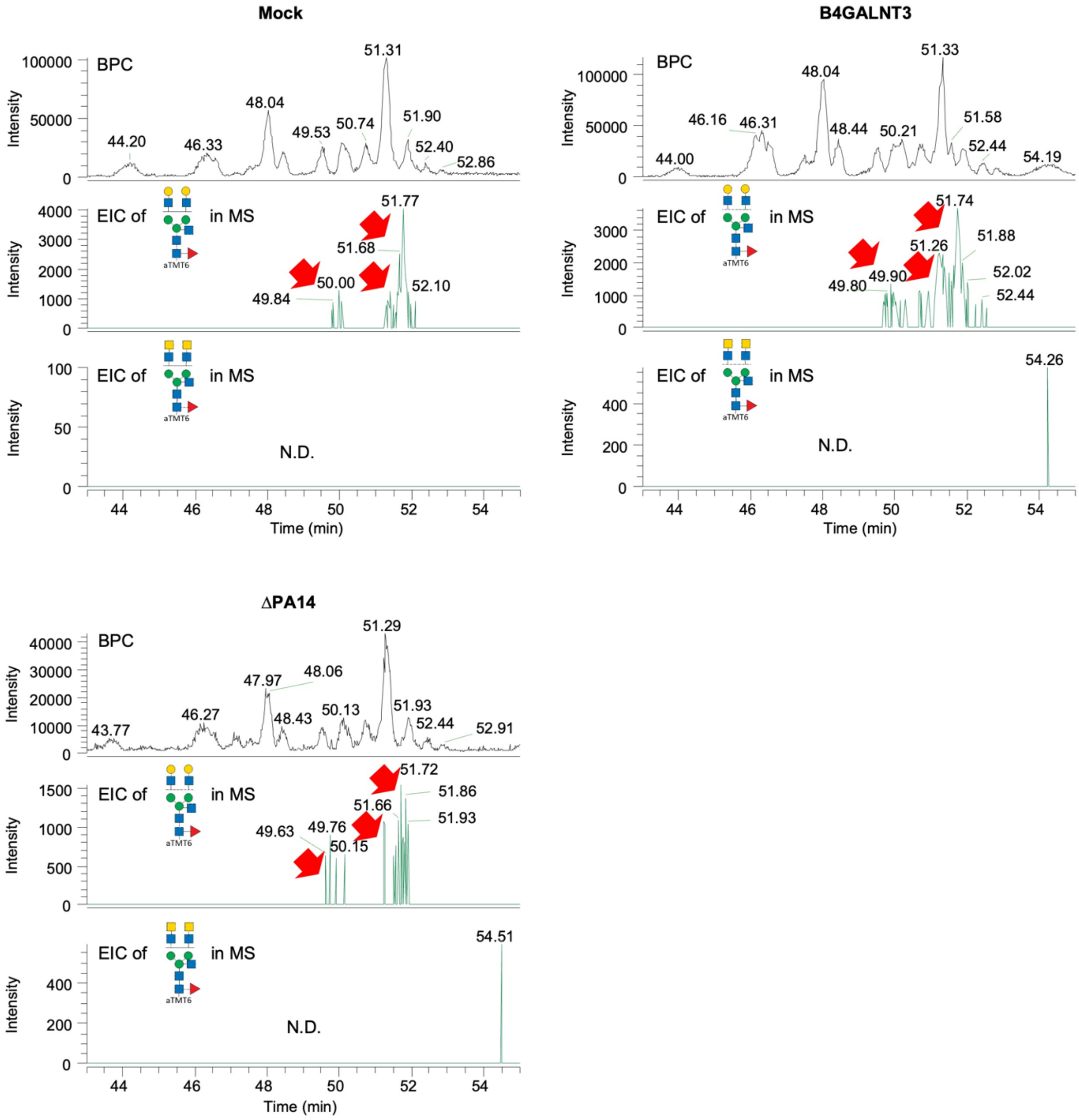

Figure S2. LC-MS analysis of *N*-glycans derived from B4GALNT3-expressing cells. EICs of the complex *N*-glycans bearing terminal LacNAc or LDN with potential bisecting GlcNAc residue from LC-MS analysis are shown. Deduced glycan structures are indicated.

Figure S3

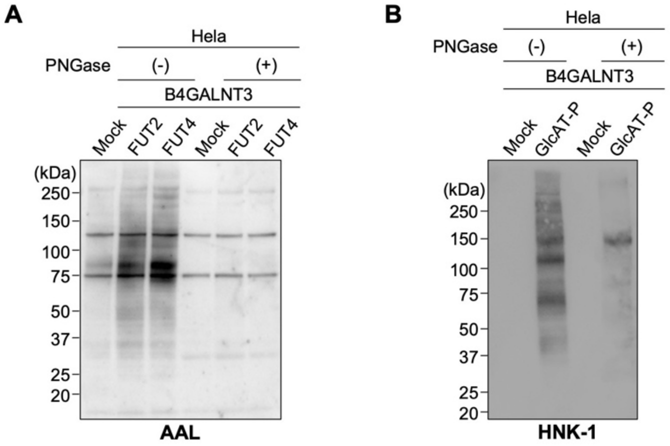

Figure S3. Fucosylation by FUT2 or FUT4 and HNK-1 biosynthesis by GlcAT-P on cellular *N*-glycans. ***A***, Hela cells were transfected with the plasmid for B4GALNT3 and the plasmid for FUT2, FUT4, or the empty vector (Mock), and the lysates were incubated with PNGaseF and analyzed by blotting with AAL lectin. ***B***, Hela cells were transfected with the plasmid for B4GALNT3 with the pIRES plasmid for both GlcAT-P and HNK-1ST or the empty vector (Mock), and the lysates were incubated with PNGaseF and analyzed by Western blotting with HNK-1 mAb.

Figure S4

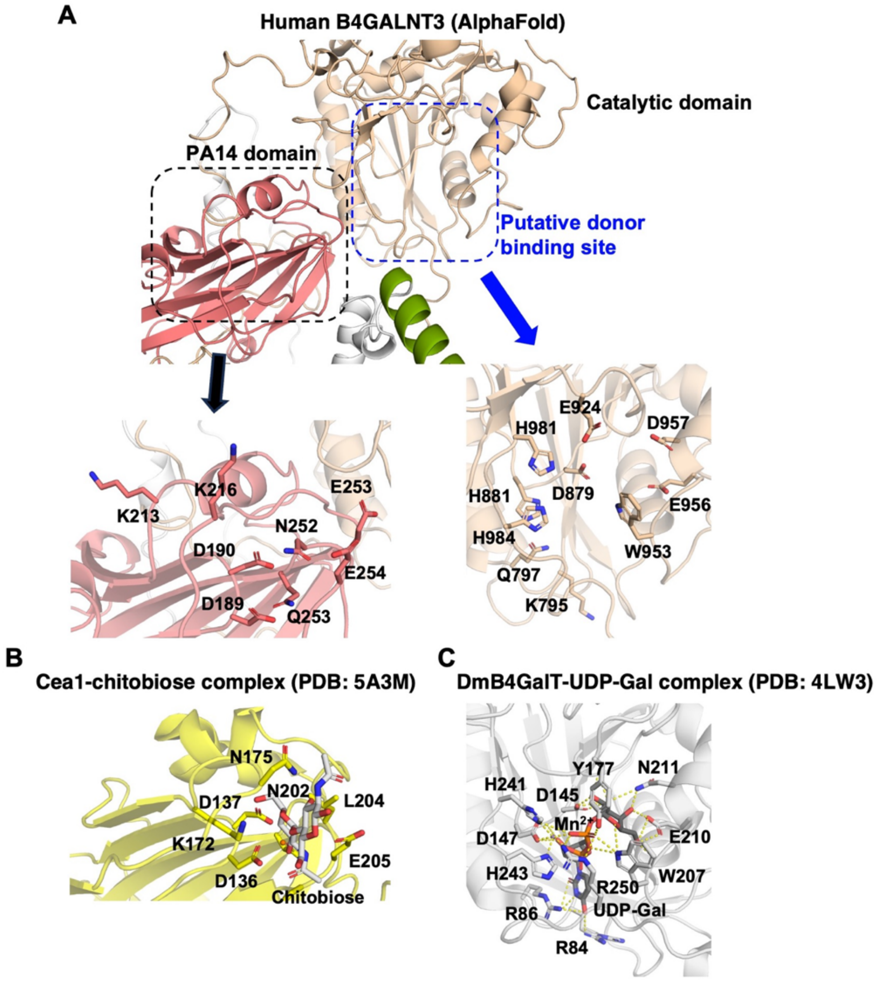

Figure S4. Structural model of B4GALNT3 and comparison with related proteins. ***A***, 3D structural model of human B4GALNT3 (UniProtKB: Q9L9W6) generated by AlphaFold2. The positions of putative donor binding site and sugar binding site are indicated with dotted boxes. Close up views of these two sites are shown in bottom right and bottom left panels. In each panel, amino acid residues which may contribute to the functions are shown as stick models and labeled. ***B***, Structural details of Cea1 PA14 domain in complex with chitobiose (PDB code: 5A3M). This figure is depicted from the same view angle as the bottom left panel of Fig. S4A. **C**, Structural details of Drosophila β4GalT in complex with donor UDP-galactose (PDB code: 4LW3). Amino acid residues which interact with UDP-galactose or manganese ion are shown in stick models and labeled. This figure is depicted from the same view angle as the bottom right panel of Fig. S4A.

Figure S5

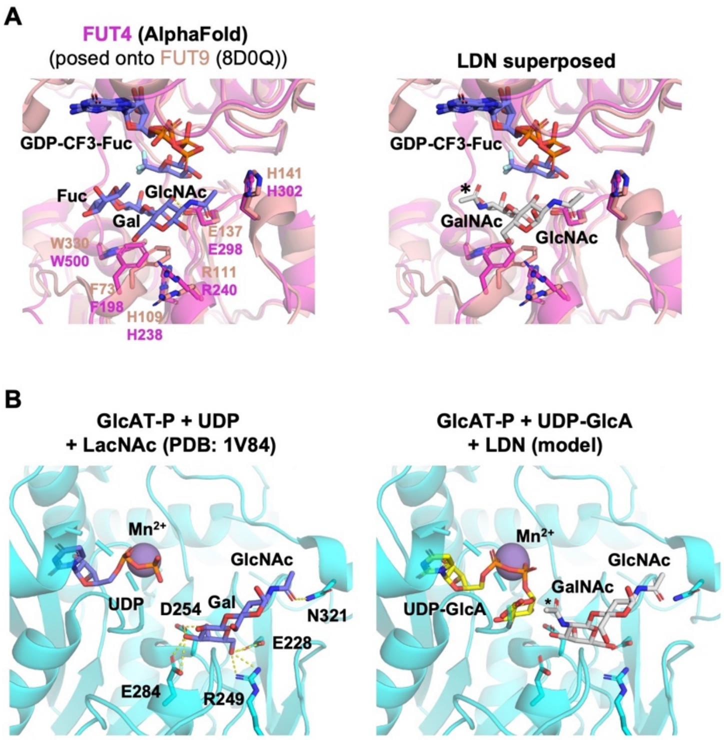

Figure S5. Docking models of glycosyltransferases for terminal modifications with LDN. ***A***, Superposition of LDN onto FUT4 and FUT9 structures. Structural superposition of hypothetical 3D structure of FUT4 generated by AlphaFold2 and crystal structure of human FUT9 in complex with donor GDP-CF3-Fucose and acceptor H-type 2 glycan (PDB: 8D0Q) was shown in the left panel. The amino acid residues close to the acceptor glycan were conserved in two fucosyltransferases. Putative position of LDN was shown in the right panel. 3D structure of LDN was retrieved from human galectin-3 in complex with LDN (PDB: 7BE3). ***B***, Superposition of LDN onto GlcAT-P structure. Crystal structure of human GlcAT-P in complexes with UDP and LacNAc (PDB: 1V84) was shown in the left panel. Putative positions of donor UDP-GlcA and LDN were shown in the right panel. Position of donor UDP-GlcA was inferred from the crystal structure of GlcAT-I in complex with UDP-GlcA (1KWS). 3D structure of LDN was also retrieved from human galectin-3 in complex with LDN (PDB: 7BE3).

Table S2.

Primers used in this study.

Primer_Name	Sequence
B4GALNT3 For	TCCACTAGTCCAGTGTGGTGGCCACCATGGGGAGCCCCCGGGCCGC
B4GALNT3 Rev	GAGCGGCCGCCACTGTGCTGGATCTACAGCGTCTTCATCTGGCGAC
ΔPA14 short For	TCCACTAGTCCAGTGTGGTGGgccaccATGGGGAGCCCCCGGGCCGC
ΔPA14 short Rev	GCTCCTGATCCAGAACCACCTCCCCAGGGGACAGGCTTGTTCCACTT
ΔPA14 long For	CCCTGGGGAGGTGGTTCTGGATCAGGAGCCAAGTTCACCATCATTGA
ΔPA14 long Rev	GAGCGGCCGCCACTGTGCTGGATCTACAGCGTCTTCATCTGGCGAC
C912A For	CCATGGTGATGAGGCTGCATGCTGGGGCCACCCCCCAGTGGC
C912A Rev	GCCACTGGGGGGTGGCCCCAGCATGCAGCCTCATCACCATGG
B4GALNT3 S60 For	ACATCACCATCACCATCACGAAGCCACCAGCTGGAGAGAACTGGCCAA
B4GALNT3 S60 Rev	CGGCCGCCACTGTGCTGGATCTACAGCGTCTTCATCTGGC
B4GALNT3 Y83 For	ACATCACCATCACCATCACGAATACCATCCCCAGAGGCTGAG
B4GALNT3 Y83 Rev	CGGCCGCCACTGTGCTGGATCTACAGCGTCTTCATCTGGC
B4GALNT3 S100 For	ACATCACCATCACCATCACGAAAGCAGTAACAGCAGCTACTT
B4GALNT3 S100 Rev	CGGCCGCCACTGTGCTGGATCTACAGCGTCTTCATCTGGC
Transferrin-myc-His For	CCACTAGTCCAGTGTGGTGGCCACCATGAGGCTCGCCGTGGGAGC
Transferrin-myc-His Rev	GAAGGGCCCTCTAGACTCGAGAGGTCTACGGAAAGTGCAGG
